# Simultaneous determination of 50 antibiotic residues in plasma by HPLC–MS/MS

**DOI:** 10.1016/j.heliyon.2024.e40629

**Published:** 2024-11-26

**Authors:** Jun Hu, Yina Ba, Zhifeng Pan, Xiaogang Li

**Affiliations:** a Shanghai Electric Power Hospital, Shanghai, China; b School of Life Science, Fudan University, Shanghai, China; c Biobank Facility, National Infrastructures for Translational Medicine, State Key Laboratory of Complex Severe and Rare Diseases, Peking Union Medical College Hospital, Chinese Academy of Medical Science and Peking Union Medical College, Beijing, China

**Keywords:** Multiclass analysis, Antibiotic residue, Liquid chromatography-tandem mass spectrometr, Qualitative analysis

## Abstract

Exposure to low doses of antibiotics in organisms may have long-term effects on host growth and brain neurochemicals, which are achieved by disrupting the composition and metabolism of gut flora. Therefore, we should pay more attention to the use and management of antibiotics to protect human health and the ecological environment. Here, we developed a method of detecting 50 antibiotic residues simultaneously in human plasma using HPLC-MS/MS. We optimized the sample pre-treatment method, chromatographic and MS parameters. The best elution buffer was 60 % acetonitrile, which ensured high recovery rate of antibiotics. The main seven kinds of antibiotics, including β-lactams, tetracyclines, macrolides, lincosamides, chloramphenicol, sulfonamides, quinolones could be detected by this method. The average recovery rate was 67.25 %–129.03 %. Analytes have been detected with limit of detection (LOD) values from 0.1 ng ml^−1^ to 5 ng ml^−1^. In brief, the method is reliable and robust for rapid screening of antibiotic residues, which was suitable and efficient to monitor antimicrobial exposure.

## 1 Introduction

Antibiotics are a class of secondary metabolites produced by microorganisms (including bacteria, fungi, actinomycetes) or higher plants and animals during their life processes, which have anti-pathogen or other activities and can interfere with the developmental functions of other living cells. The bacteriostatic or bactericidal effects of antibiotics and other antimicrobial agents are mainly targeted at the mechanism of "bacteria have but humans (or other animals and plants) do not", including four major mechanisms: inhibiting bacterial cell wall synthesis, enhancing bacterial cell membrane permeability, interfering with bacterial protein synthesis, and inhibiting bacterial nucleic acid replication and transcription. There are many types of antibiotics, and commonly used antibiotics in clinical practice include extracts from microbial culture broths and chemically synthesized or semi-synthesized compounds. Antibiotics can be used to treat or inhibit pathogenic microbial infections [1,2]. Besides, it is frequently used in animal husbandry to promote growth of food-producing animals [3]. In order to protect human consumers from harmful effects of antibiotic residues, governmental organizations established maximum residue limits (MRLs) in foodstuff [4]. However, research by Martin Blaser and John Bieneistock has found that early exposure to low doses of antibiotics (below MRLs) in organisms may have long-term effects on host growth and brain neurochemicals, which are achieved by disrupting the composition and metabolism of gut flora. Therefore, we should pay more attention to the use and management of antibiotics to protect human health and the ecological environment [[5], [6], [7], [8]].

Many methods of antibiotic residues analysis have been developed. Microbiological assays are easy to perform, but the process typically takes 2–3 days and has relatively low specificity and sensitivity [[9], [10], [11], [12], [13], [14]]. Alternatively, enzyme-linked immunosorbent assay (ELISA) can provide rapid analysis, but this method needs multi-steps including elution and addition, making it difficult for non-professionals to use effectively in on-site (or outside the laboratory) situations [[15], [16], [17]]. Lateral flow immunoassay (LFIA) is specifically designed for simplicity and speed, making it an effective method for rapid point-of-care testing [[18], [19]]. High-performance liquid chromatography and liquid chromatography-tandem mass spectrometry (HPLC-MS/MS) method has become an important trend, which can identify and quantify as many antibiotics as possible in plasma in one run. This method is efficient, accurate, and sensitive, and can quickly separate and identify various antibiotics.

This paper is a complementary system for our europium chelate-labeled lateral flow assay system, so the linear range of this method was set to cover the test strip detection limit [20]. The purpose of this paper is to develop a method for simultaneously detecting 50 antibiotics in seven categories (β-lactams, eight quinolones, eight sulfonamides, four tetracyclines, three macrolides, and one lincosamide). The pre-treatment and extraction methods were optimized, and the method was extensively validated at Shanghai Electric Power Hospital.

## 2 Material and methods

### 2.1 Ethics and study design

Healthy were enrolled from Shanghai Electric Power Hospital between March 2022 and June 2023. The protocols and methods are implemented according to recommendation of the Shanghai Electric Power Hospital institutional committee to protect human subjects. This study was reviewed and approved by institutional committee that protects human subjects from Shanghai Electric Power Hospital with the approval number:2022–02175, dated 2022.6. All participants provided written informed consent to participate in the study and for their data to be published.

### 2.2 Reagents and chemicals

Penicillin, Ampicillin, Oxacillin, Flucloxacillin, Cloxacillin, Mezlocillin, Ceftiofur, Cefotaxime, Cefradine, Meropenem, Cephalothin, Cefoperazone, Amoxicillin, Cefadroxil, Cefmetazole, Cefaclor, Cefazolin, Cefalexin, Sultamicillin, Ceftazidime, Sulfadimidine, Sulfadiazine, Sulfapyridine, Sulfathiazloe, Sulfacetamide Sodium, Sulfamethizole, Sulfamethoxipyridazine, Sulfamonomethioxine, Sulfachloropyridazine, Levofloxacin, Ciprofloxacin, Fleroxacin, Lomefloxacin, Pazufloxacin, Pefloxacin, Sparfloxacin, Flumequine, Antofloxacin, Enrofloxacin, Nadifloxacin, Norfloxacin, Enoxacin, Tetracycline, Oxytetracycline, Chlorotetracycline, Lincomycin, Clindamycin, Chloramphenicol, Florfenicol, Thiamphenicol were purchased from the National Institutes for Food and Drug Control (Beijing, China). Oasis-HLB solid phase extraction cartridges (60 mg, 3 cm^3^) were obtained from Waters (Milford, MA). Stock solutions were set as 1 mg/mL and diluted to gradient concentrations for test.

### 2.3 Preparation procedures

The pre-processing process includes the following steps. (1) Activation of Oasis HLB μEluting Plate: Add 200 μL of methanol to the small pores of the Oasis HLB μEluting Plate, filter with suction; rinse with 200 μL of ultrapure water and filter twice; discard the filtrate. (2) Sample processing: Take a dry blood smear sample, cut it along the bloodstain color or dotted line edge, and try to cut it into pieces. Add 250 μL of 0.01 mol PBST solution (0.5 % Tween20 in PBS buffer), shake and mix for 1 h (3) Sample extraction: Take 200 μL of solution from step 2 (equivalent to 20 μL of whole blood sample), add it to the activated Oasis HLB μEluting Plate wells, and filter the solution until the liquid in the wells is drained. Discard the filtrate. (4) Rinse: Add 100 μL of ultrapure water for rinsing and suction filtration, and then repeat the rinsing and suction filtration once more until the liquid in the well is drained, discarding the filtrate. (5) Elution: Add 100 μL of 60 % acetonitrile for elution and filter with suction. Repeat the elution and filtration process once more until the liquid in the well is drained. Collect the two elution solutions from the elution step using a 96-well plate. (6) Injection: The above eluent was directly injected, with an injection volume of 5 μL.

### 2.4 LC-MS/MS conditions

The sample analyses were performed using a Waters ACQUITY UPLC H-Class FTN UPLC system coupled with a Waters Xevo TQD triple quadrupole mass spectrometer equipped with an electrospray ionization (ESI) source. Chromatographic separation were achieved using an Waters CORTECS T3 column (2.7 μm, 2.1 × 100 mm). The column temperature was kept at 35 °C. The analytes were separated with a mobile phase consisting of 0.1 % formic acid in water (eluent A) and acetonitril (eluent B) at a flow rate of 0.4 mL/min. The gradient program was set as follows: 0 min 5 % B, 0.2 min 10 % B,3.5 min 40 % B, 5.5 min 55 % B, 7.5 min 90 % B,7.5–18 min 90 % B, and 18.1–20 min 5 % B. The flow rate was set at 0.4 mL/min, and the injection volume was 5 μl. The samples were kept in an autosampler at 15 °C.

**Fig. 1:**
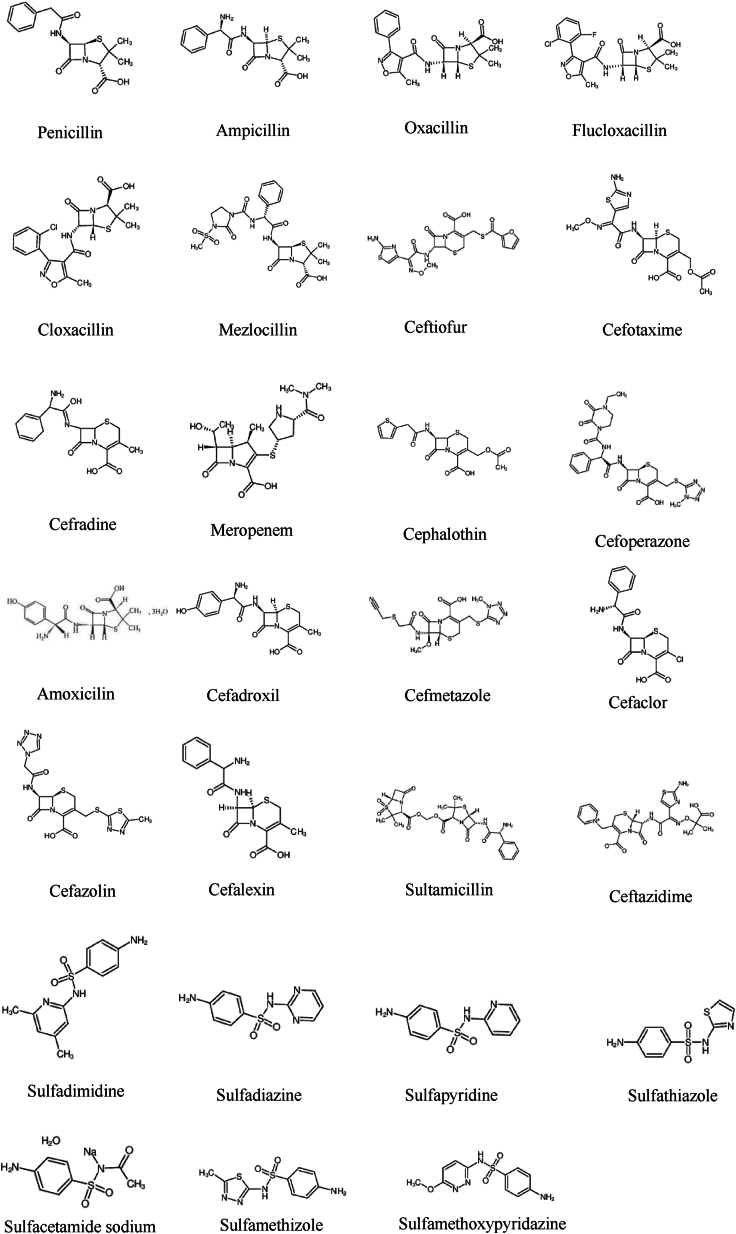

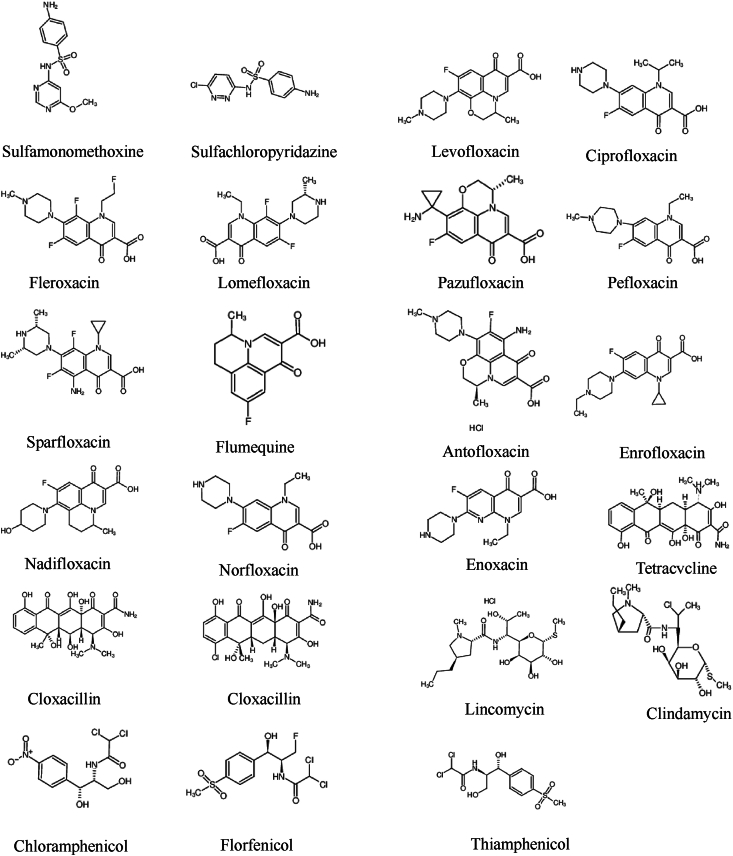
Chemical structure of antibiotics.

The mass spectrometry analyses were carried out using a Waters Xevo TQD.The instrument was operated using an electrospray (ESI) source in positive and negative ion (ESI+/ESI -) switching mode with following settings: capillary voltage 3.0 kV+/2.5 kV-, ion source temperature 150 °C, desolvent gas temperature 550 °C, desolvent gas flow rate 1000 L/h, and cone hole gas flow rate 50 L/h. The collection and analysis were performed using the multiple reaction monitoring mode (MRM) (see Fig. 1).

## 3 Results

### 3.1 Optimization of sample pre-treatment methods

**Fig. 2:**
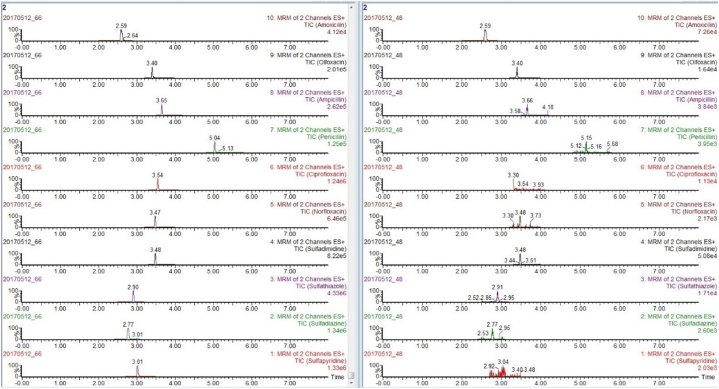
Optimization of sample pre-treatment method.

A comparison was made between the traditional acetonitrile precipitation and nitrogen-blowing reconstitution method after centrifugation and the Oasis HLB solid phase extraction method. The results showed that the purification effect and peak area of the Oasis HLB SPE method were better than those of the protein precipitation nitrogen-blowing method, and it was less prone to cross-contamination (Fig. 2). The HLB SPE method (Fig. 2, left) showed higher signal-to-noise ratio compared to the protein precipitation method.

The investigation of eluent showed that methanol was prone to elute phospholipids and other substances. Therefore, aqueous acetonitrile solutions with different concentration gradients (100 %, 80 %, 60 %) were selected for elution. We used the signal-to-noise ratio and recovery rate of the MassLynx software to compare to chromatograms of the two extraction methods. The results showed that the high organic phase ratio of acetonitrile solution would produce higher background noise. The effect of 60 % acetonitrile was the best, which ensured high recovery rate of antibiotics.

### 3.2 Optimization of chromatographic and MS parameters

The BEH C18 (1.7 μm, 2.1 × 50 mm) and CORTECS T3 (2.7 μm, 2.1 × 100 mm) chromatographic columns of the Waters ACQUITY UPLC system were investigated separately. The results showed that the T3 chromatographic column had a large polarity span and had better retention for amoxicillin and other more polar compounds. The selection of mobile phase showed that when methanol was used as the solvent, the antibiotics could be completely separated and methanol could improve the peak shape. Therefore, methanol was selected as the mobile phase.

**Fig. 3:**
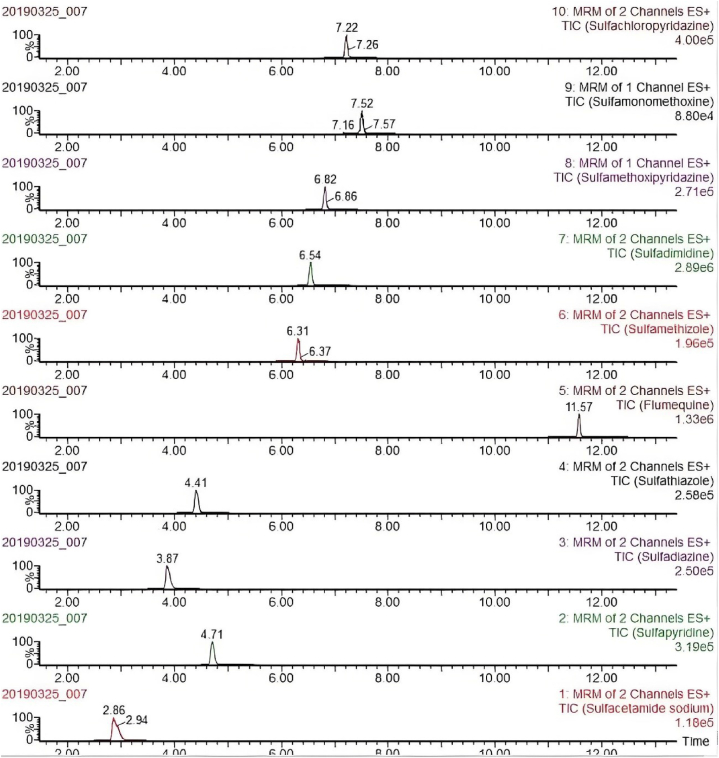

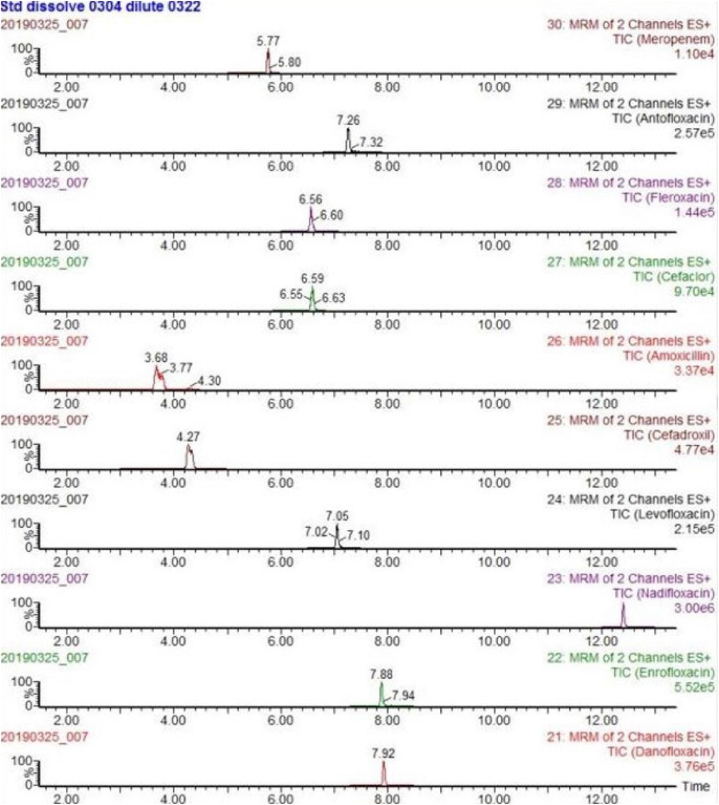

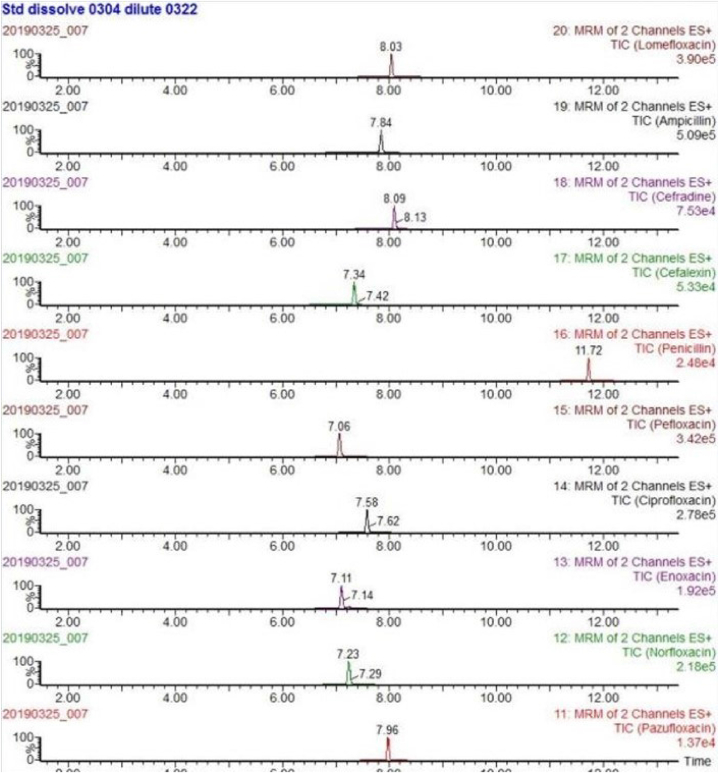

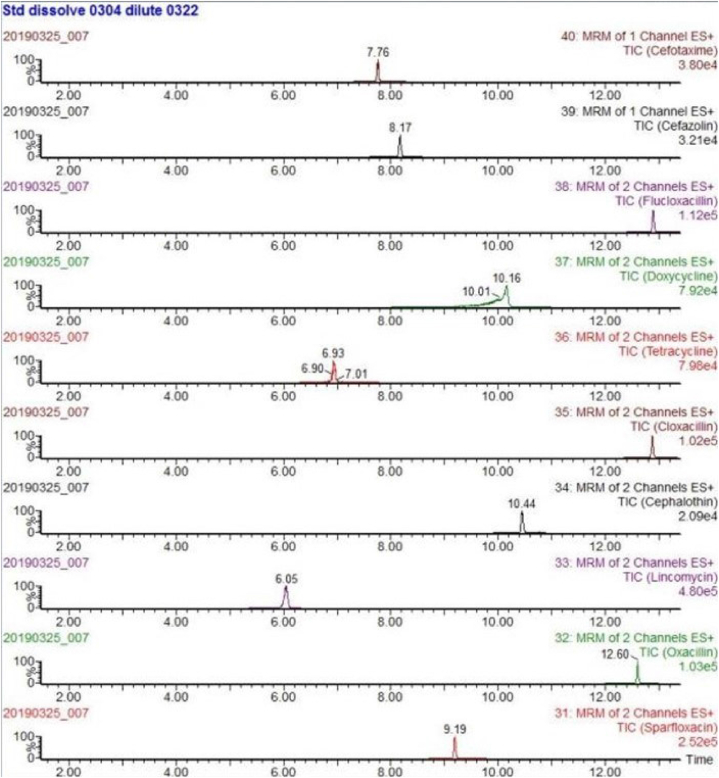

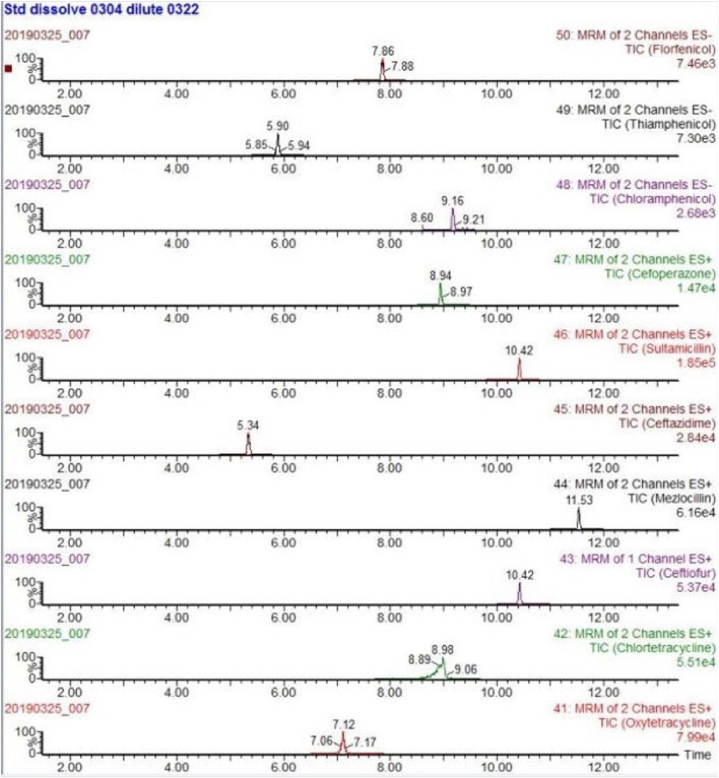
Total ion chromatogram (TIC) of different kinds of sulfonamide.

**Fig. 4:**
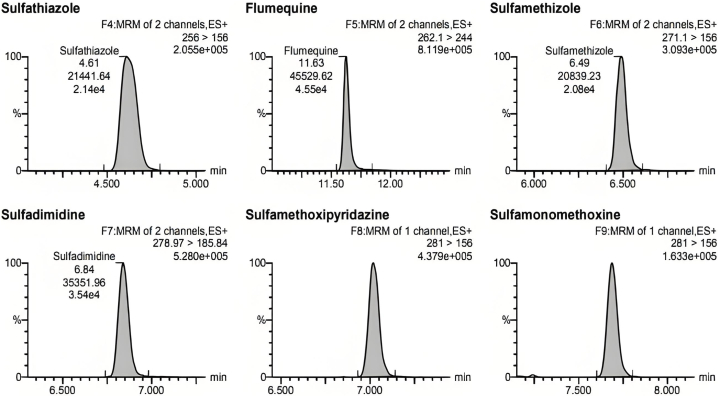
Chromatogram of different kinds of sulfonamide.

Due to the different mass spectrometric behaviors of antibiotics in the seven major groups, the majority of antibiotic molecules have strong signals in positive ion mode (ESI+), while chloramphenicol antibiotics have the opposite effect, with almost no detectable signals in positive ion mode, making them suitable for monitoring in negative ion mode (ESI-). Therefore, this method chooses the MRM method with positive and negative ion modes for simultaneous multi-reaction monitoring, allowing for the analysis of all target antibiotics in one step, reducing analysis time. For MRM technology, the key step was to detect the specific parent ion, then only the selected specific parent ion was collision-induced and only the selected specific daughter ion was collected by mass spectrum detector (Fig. 3). From the MS/MS spectrum, the two most intense transitions of each compound were selected for operation in sMRM mode.one transition was used for quantification (marked with “∗”), and the other was used for qualitative confirmation. We also optimized the mobile phase gradient to stagger the retention time of the different antibiotics, there was no simultaneous peak in the elution phase (Fig. 4). For example, the different kinds of sulfonamide were detected simultaneously without mutual interference (see Table 1).

**Table 1:** MS parameters for 50 antibiotics.

Analyte	Ionization mode	Parent ion (*m*/*z*)	Daughter ion (*m*/*z*)	retention time (min)	cone voltage (V)	Collision energy (V)
Penicillin	**ESI+**	334.97	159.91	11.74	24	15
	**ESI+**	334.97	175.97		24	15
Ampicillin	**ESI+**	350.2	106	7.52	30	18
	**ESI+**	350.2	160		30	12
Oxacillin	**ESI+**	402.2	160	12.63	30	12
	**ESI+**	402.2	243.1		30	15
Flucloxacillin	**ESI+**	454.03	114.03	12.93	24	46
	**ESI+**	454.03	160.05		24	12
Cloxacillin	**ESI+**	436.2	160	12.91	27	15
	**ESI+**	436.2	277.1		27	15
Mezlocillin	**ESI+**	540.1	253.07	11.55	32	32
	**ESI+**	540.1	296.04		32	20
Ceftiofur	**ESI+**	524.2	241.1	10.53	35	16
	**ESI+**	–	–		–	–
Cefotaxime	**ESI+**	456.1	396.2	7.78	30	10
	**ESI+**	–	–		–	–
Cefradine	**ESI+**	350.16	157.99	7.88	22	8
	**ESI+**	350.16	176		22	12
Meropenem	**ESI+**	384.16	113.99	5.56	30	26
	**ESI+**	384.16	141.09		30	14
Cephalothin	**ESI+**	419.06	315.04	10.45	32	18
	**ESI+**	419.06	359.04		32	16
Cefoperazone	**ESI+**	668.1	165.07	8.95	34	28
	**ESI+**	668.1	525.94		34	18
Amoxicilin	**ESI+**	366.2	114	3.13	27	20
	**ESI+**	366.2	349.1		27	8
Cefadroxil	**ESI+**	364.1	113.98	3.86	18	20
	**ESI+**	364.1	207.96		18	10
Cefmetazole	**ESI+**	493.97	349.8	7.62	30	16
	**ESI+**	493.97	333.8		30	18
Cefaclor	**ESI+**	368.03	105.99	6.37	24	22
	**ESI+**	368.03	173.98		24	14
Cefazolin	**ESI+**	455.1	323.2	8.14	24	15
	**ESI+**	–	–		–	–
Cefalexin	**ESI+**	348.13	157.93	7.07	20	8
	**ESI+**	348.13	173.91		20	12
Sultamicillin	**ESI+**	595.1	106.06	9.75	34	46
	**ESI+**	595.1	114.05		34	40
Ceftazidime	**ESI+**	547.07	166.95	5.34	26	22
	**ESI+**	547.07	467.8		26	12
Sulfadimidine	**ESI+**	278.97	91.97	6.84	40	34
	**ESI+**	278.97	185.84		40	18
Sulfadiazine	**ESI+**	251	92	4.08	30	27
	**ESI+**	251	156		30	15
Sulfapyridine	**ESI+**	250	108	4.97	33	25
	**ESI+**	250	156		33	16
Sulfathiazole	**ESI+**	256	92	4.63	31	25
	**ESI+**	256	156		31	15
Sulfacetamide sodium	**ESI+**	215	108	2.95	25	18
	**ESI+**	215	156		25	12
Sulfamethizole	**ESI+**	271.1	92	4.63	30	25
	**ESI+**	271.1	156		30	15
Sulfamethoxipyridazine	**ESI+**	281	156	7.02	35	22
	**ESI+**	–	–		–	–
Sulfamonomethoxine	**ESI+**	281	156	7.69	35	22
	**ESI+**	–	–		–	–
Sulfachloropyridazine	**ESI+**	285.1	92	7.37	32	28
	**ESI+**	–	–		–	–
Levofloxacin	**ESI+**	362.03	260.95	6.94	42	32
	**ESI+**	362.03	318.04		42	32
Ciprofloxacin	**ESI+**	332.1	288.1	7.46	42	18
	**ESI+**	332.1	314.1		42	22
Fleroxacin	**ESI+**	370.4	269.3	6.45	30	25
	**ESI+**	370.4	326.3		30	20
Lomefloxacin	**ESI+**	352.1	265.1	7.93	39	22
	**ESI+**	352.1	308.1		39	16
Pazufloxacin	**ESI+**	319.16	284.04	7.86	22	16
	**ESI+**	319.16	301.99		22	10
Pefloxacin	**ESI+**	334.1	290.1	6.97	42	19
	**ESI+**	334.1	316.1		42	19
Sparfloxacin	**ESI+**	393.3	292.3	9.1	30	25
	**ESI+**	393.3	349.3		30	20
Flumequine	**ESI+**	262.1	202	11.62	35	35
	**ESI+**	262.1	244		35	15
Antofloxacin	**ESI+**	377.22	276.07	7.15	40	28
	**ESI+**	377.22	333.14		40	20
Enrofloxacin	**ESI+**	360.17	244.96	7.8	42	28
	**ESI+**	360.17	316.01		42	18
Nadifloxacin	**ESI+**	361.16	257.06	12.46	40	38
	**ESI+**	361.16	343.07		40	22
Norfloxacin	**ESI+**	320.1	233	7.1	40	25
	**ESI+**	320.1	276.1		40	20
Enoxacin	**ESI+**	321.1	232	6.97	40	30
	**ESI+**	321.1	303.1		40	35
Tetracycline	**ESI+**	445.03	153.93	7	32	32
	**ESI+**	445.03	409.95		32	32
Oxytetracycline	**ESI+**	460.65	426.22	7.21	34	18
	**ESI+**	460.65	444.23		34	18
Chlortetracycline	**ESI+**	479.26	154.12	9.02	36	26
	**ESI+**	479.26	444.19		36	20
Lincomycin	**ESI+**	407.09	125.96	5.88	45	34
	**ESI+**	407.09	359.08		45	20
Clindamycin	**ESI+**	425.17	126.07	10.57	42	26
	**ESI+**	425.17	377.04		42	18
Chloramphenicol	**ESI+**	320.84	151.78	9.25	32	18
	**ESI+**	320.84	256.83		32	13
Florfenicol	**ESI+**	355.9	184.95	7.93	26	20
	**ESI+**	355.9	335.81		26	8
Thiamphenicol	**ESI+**	353.9	184.96	5.97	36	22
	**ESI+**	353.9	289.86		36	12

### 3.3 Determination of antibiotics

**Fig. 5:**
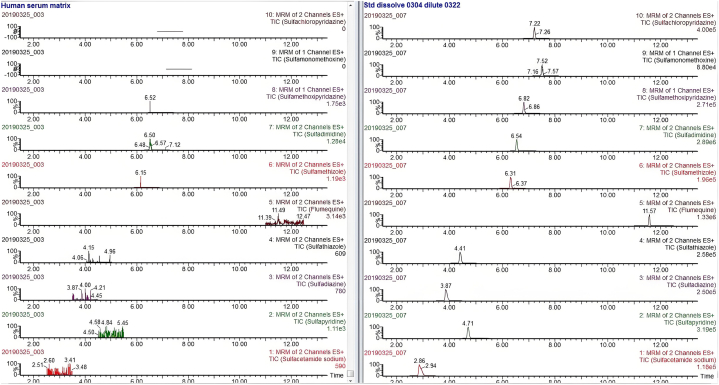
Ion signal values after addition of sulfonamides

The samples were prepared using the Oasis HLB solid phase extraction method. This project is a complementary system for the europium chelate-labeled lateral flow assay system, so the linear range of this method was set to cover the test strip detection limit [20]. In our previous study, we used broadly specific penicillin-binding protein (PBP) that can theoretically detect all kinds of b-lactams. But, it could not detect other kinds of antibiotics using the same strip. The main advantage of this paper was the possibility of detecting multiple analytes in the same run. In this study, we prepared standard material by adding gradient concentration of specific antibiotics to mixture of 300 μL negative sera from 30 participants after physical examination in our hospital. After addition of standard products to the human sera matrix, the difference in signal values is more than 2 orders of magnitude, so matrix interference is excluded, indicating good specificity (Fig. 5). In this project, we defined LOD as concentration causing signal to noise (S/N) ≥3 times. Lower Limit of quantitation (LLOQ) refers to the lower limit of the quantifiable concentration that can be achieved using this method. LLOQ was the concentration causing signal to noise (S/N) ≥10 times. The accuracy of the analytical method was determined following these steps: add standard material to form low, medium, and high concentrations of spiked test solutions (n = 3); analyze each of the 3 parallel samples and determine the actual concentration of the target element; calculate the recovery rate of each sample, average recovery rate and relative standard deviation (RSD) for each concentration to evaluate the accuracy of the method. We also repeated 6 parallel samples for each concentration to evaluated the reliability of this analytical methods, and the recovery range was between 67.25 % and 129.03 % (Table 2).

**Table 2:** Standard curve.

Analyte	Linear range (ng/mL)	LOD (ng/mL)	LLOQ (ng/mL)	LOD in serum(ng/mL)	Stability at 4 °C	Accuracy	Recovery range
Penicillin	1–100	0.2	0.2	1	93.30 %	97.50 %	95.74 %–102.59 %
Ampicillin	1–100	0.1	0.2	1	99.10 %	96.80 %	84.16 %–109.40 %
Oxacillin	1–100	0.1	0.2	1	100.40 %	104.30 %	97.96 %–100.67 %
Flucloxacillin	1–100	0.1	0.2	1	99.90 %	106.20 %	86.73 %–98.69 %
Cloxacillin	1–100	0.1	0.2	1	99.80 %	80.30 %	96.21 %–122.03 %
Mezlocillin	1–100	0.2	0.2	1	98.90 %	99.90 %	102.27 %–103.09 %
Ceftiofur	1–100	0.1	0.2	1	95.70 %	95.90 %	94.56 %–111.27 %
Cefotaxime	1–100	0.1	0.2	1	96.10 %	95.70 %	88.44 %–129.03 %
Cefradine	1–100	0.1	0.2	1	92.50 %	88.40 %	90.53 %–100.32 %
Meropenem	1–100	0.2	0.2	1	91.70 %	91.70 %	85.98 %–117.13 %
Cephalothin	2–200	0.4	0.4	2	91.80 %	95.30 %	90.5 %–107.80 %
Cefoperazone	2–200	0.4	0.4	2	90.30 %	107.40 %	79.9 %–112.90 %
Amoxicillin	5–500	NQ	1	5	98.90 %	96.90 %	70.57 %–101.21 %
Cefadroxil	5–500	NQ	1	5	103.40 %	83.00 %	67.25 %–103.92 %
Cefmetazole	5–500	0.5	1	5	102.70 %	104.30 %	91.37 %–102.17 %
Cefaclor	5–500	1	5	5	93.20 %	99.70 %	92.84 %–110.07 %
Cefazolin	5–500	2	5	5	89.90 %	96.20 %	95.80 %–99.50 %
Cefalexin	5–500	2	5	5	92.5	91.50 %	86.36 %–105.94 %
Sultamicillin	10–1000	5	10	10	89.20 %	95.30 %	66.80 %–99.30 %
Ceftazidime	10–1000	5	10	10	93.20 %	100.30 %	79.72 %–103.90 %
Sulfadimidine	1–100	0.1	0.2	1	91.00 %	100.00 %	86.51 %–107.47 %
Sulfadiazine	1–100	0.1	0.2	1	104.60 %	86.50 %	92.08 %–100.20 %
Sulfapyridine	1–100	0.1	0.2	1	96.60 %	85.40 %	90.81 %–111.77 %
Sulfathiazloe	1–100	0.1	0.2	1	102.40 %	82.20 %	92.48 %–105.30 %
Sulfacetamide Sodium	1–100	0.1	0.2	1	98.10 %	93.00 %	94.00 %–114.37 %
Sulfamethizole	1–100	0.1	0.2	1	94.60 %	85.90 %	94.70 %–111.50 %
Sulfamethoxipyridazine	1–100	0.1	0.2	1	101.90 %	91.30 %	80.84 %–108.43 %
Sulfamonomethioxine	1–100	0.1	0.2	1	97.40 %	89.20 %	95.29 %–113.83 %
Sulfachloropyridazine	1–100	0.1	0.2	1	94.20 %	93.90 %	99.29 %–110.79 %
Levofloxacin	1–100	0.1	0.2	1	99.80 %	100.40 %	89.55 %–101.50 %
Ciprofloxacin	1–100	0.1	0.2	1	97.60 %	81.40 %	96.44 %–106.09 %
Fleroxacin	1–100	0.1	0.2	1	91.60 %	82.20 %	96.02 %–102.92 %
Lomefloxacin	1–100	0.1	0.2	1	100.00 %	85.80 %	93.74 %–97.63 %
Pazufloxacin	1–100	0.1	0.2	1	95.60 %	104.20 %	84.69 %–103.64 %
Pefloxacin	1–100	0.1	0.2	1	96.50 %	95.30 %	73.57 %–90.49 %
Sparfloxacin	1–100	0.1	0.2	1	93.10 %	99.20 %	92.41 %–98.90 %
Flumequine	1–100	0.1	0.2	1	94.50 %	85.90 %	99.43 %–107.24 %
Antofloxacin	1–100	0.1	0.2	1	104.50 %	81.30 %	90.02 %–114.03 %
Enrofloxacin	1–100	0.1	0.2	1	93.90 %	109.70 %	77.07 %–96.40 %
Nadifloxacin	2–100	0.1	0.2	2	93.10 %	90.60 %	93.19 %–113.93 %
Norfloxacin	2–200	0.4	0.4	2	90.80 %	84.00 %	99.35 %–113.65 %
Enoxacin	2–200	0.4	0.4	2	97.40 %	109.90 %	99.69 %–112.12 %
Tetracycline	1–100	0.1	0.2	1	92.70 %	97.70 %	75.61 %–97.62 %
Oxytetracycline	1–100	0.1	0.2	1	103.50 %	103.70 %	91.01 %–101.32 %
Chlorotetracycline	1–100	0.1	0.2	1	98.60 %	107.50 %	91.66 %–115.23 %
Lincomycin	1–100	0.1	0.2	1	94.70 %	105.20 %	76.07 %–99.73 %
Clindamycin	1–100	0.1	0.2	1	95.70 %	100.20 %	90.28 %–107.40 %
Chloramphenicol	1–100	0.2	0.2	1	102.40 %	80.80 %	80.87 %–109.11 %
Florfenicol	5–500	1	5	5	97.70 %	103.60 %	79.20 %–91.75 %
Thiamphenicol	10–1000	2	10	10	93.10 %	86.00 %	90.61 %–103.50 %

### 3.4 Application of the method to real samples

We screened the serum samples from some volunteers. The basal information was collected from Shanghai Electric Power Hospital. We found some patients showed positive results of antibiotics (Table 3). Most of the samples detected are physical examination samples without history of medication use. If drug concentration of sample beyond the upper limit of the linear range, the sample would be diluted according to the protocol and re-analyzed. The source of antibiotics should be fully investigated. Suarez et al. assessed the risk of transfer of residues to edible matrices after exposure to cross-contaminated feed using a LC-MS/MS method for the quantitative analysis of four antibiotics in pig tissues and plasma [21]. Wang H et al. selected 586 school children aged 8–11years from Shanghai in 2013, total urinary concentrations (free and conjugated) of 21 common antibiotics from six categories (macrolides, β-lactams, tetracyclines, fluoroquinolones, sulfonamides, and phenicols) were measured by UPLC MS/MS. All 21 antibiotics were found in urines with the overall detection frequency of 79.6 % [22].

**Table 3:** Detected antibiotics in the Participants of this study.

Participants(n)	Category	Detected Antibiotic	Positive (n)
6199	β-lactams	Amoxicilin, Penicillin, Ampicillin, Oxacillin, Cloxacillin, Cefradine, Cefalexin, Mezlocillin, Ceftazidime, Meropenem, Meropenem,	52
	quinolones	Levofloxaci, Ciprofloxacin, Norfloxacin, Antofloxacin, Enrofloxacin, Pefloxacin, Enoxacin	24
	sulfonamides	Sulfadimidine, Sulfadiazine, Sulfamethoxipyridazine	2
	tetracyclines	Doxycycline	1
	chloramphenicol	Chloramphenicol	1
	lincosamides	–	–
	macrolides	Clindamycin	1

## 4 Conclusions

This project has successfully developed a mass spectrometry detection method that can simultaneously detect 50 types of antibiotics. In addition, we applied and validated the method on 80 patient samples from Shanghai Power Hospital, demonstrating its good application value. The application of this mass spectrometry method is widespread. It can not only be used to monitor antibiotic residues but also to monitor blood drug concentrations, which is of great significance for guiding clinical rational drug use. At the same time, the method has high sensitivity and good specificity, accurately detecting different types of antibiotics, reducing cumbersome steps and manual errors in traditional detection methods, and improving detection efficiency and accuracy. In terms of application validation, we tested the method on 80 patient samples from Shanghai Power Hospital. The recovery range was between 67.25 % and 129.03 %, the reason may be that some specific antibiotics have a different behavior depending on the pH, thus influencing the recovery values of the method [23]. The results showed that the method could accurately detect antibiotic residues in the patient samples, demonstrating its feasibility and reliability in practical applications.

In summary, the successful implementation of this project provides a new solution for monitoring antibiotic residues. The method has the advantages of high sensitivity, good specificity, and high efficiency, accurately detecting different types of antibiotics, and is suitable for monitoring and managing clinical rational drug use. We believe that the application of this method will help improve the quality and safety of medical care.

## CRediT authorship contribution statement

**Jun Hu:** Investigation, Data curation. **Yina Ba:** Validation, Methodology. **Zhifeng Pan:** Writing – original draft, Formal analysis, Data curation. **Xiaogang Li:** Writing – review & editing, Writing – original draft, Methodology, Investigation, Formal analysis, Conceptualization.

## Data availability statement

Data included in article/supp. material/referenced in article.

## Funding

This work was supported by the National High Level Hospital Clinical Research Funding (2022-PUMCH-A-055; 2022-PUMCH-A-263; 2023-PUMCH-F-004).

## Declaration of competing interest

The authors declare that they have no known competing financial interests or personal relationships that could have appeared to influence the work reported in this paper.
